# Late-onset mycotic aneurysm of brachial artery secondary to infective endocarditis: case report

**DOI:** 10.11604/pamj.2022.43.93.27874

**Published:** 2022-10-20

**Authors:** Hammam Rasras, Taha Abu Al Tayef, Maryem Assmati, Omar El Mahi, Adnane Benzirar, Noha El Ouafi

**Affiliations:** 1 Department of Cardiology, Mohammed VI University Hospital of Oujda, Mohammed First University of Oujda, Oujda, Morocco,; 2 Department of Vascular Surgery, Mohammed VI University Hospital of Oujda, Mohammed First University of Oujda, Oujda, Morocco,; 3 Laboratory of Epidemiology, Clinical Research and Public Health, Faculty of Medicine and Pharmacy, Mohammed the First University of Oujda, Oujda, Morocco

**Keywords:** Infective endocarditis, mycotic aneurysm, brachial artery, *Streptococcus SPP*, case report

## Abstract

Infective endocarditis remains a dreaded and severe disease because of its course, its complications, its need for good antibiotic management, as well as its morbidity. Peripheral mycotic aneurysm is a rare complication of infective endocarditis. We report the case of a 62-year-old female patient who presented with Streptococcus SPP infective endocarditis treated by antibiotics. Two months later, she presented with a mycotic aneurysm of the right brachial artery with the same germ, we have performed a brachial artery ligation, then a complete revascularization by graft of the saphenous vein. Through this case, we should not forget that the lesional assessment is always necessary to avoid missing serious complications that could threaten the functional prognosis, even vital.

## Introduction

Infective endocarditis is a rare affection, of polymorphic expression and very severe due to its cardiac and extra-cardiac complications. Vascular complications are presented especially by embolization and mycotic aneurysm, which are uncommon and happened in 2.5 - 10% cases [1]. mycotic aneurysm results from septic embolization of vegetation to vasa vasorum of arteries or to the intraluminal space. It is a major cause of morbidity and mortality although a well-adapted antibiotic therapy. All arterial territories can be affected: cerebral arteries, aorta, digestive arteries and rarely peripheral arteries [2]. Infective endocarditis is a lethal disease if not promptly treated with antibiotics, either in association with surgery or not. The treatment consists the infection´s eradication by excision the aneurysm and maintenance of adequate distal circulation. We report the case of a 62-year-old female patient who presented with a Late-onset Mycotic Aneurysm of Brachial Artery complicating an infective endocarditis well treated by antibiotics.

## Patient and observation

**Patient information:** a 62-year-old female patient, with a past history of recurrent tonsillitis in childhood, non-smoker, non-alcoholic.

**Clinical findings:** the patient presented with an asthenia with fever for 1 month. On physical examination, a pan-systolic murmur was heard at the apex radiating to the axilla with a poor oral hygiene. Respiratory, abdominal, neurological and skin examinations were unremarkable.

**Timeline of current episode:** historical and current information from this episode of care organized as a timeline.

**Figure 1:**
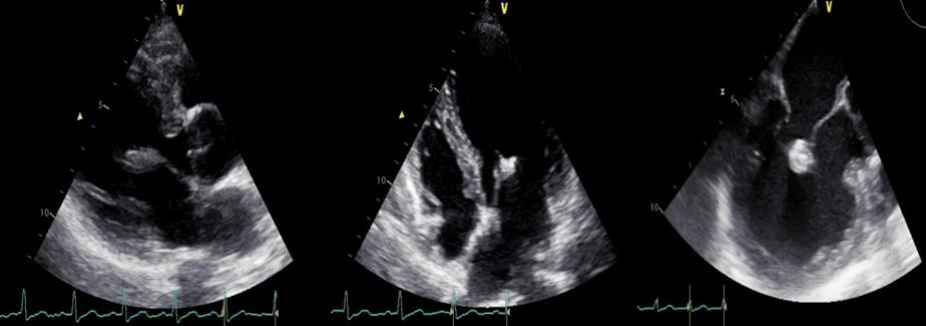
couple TTE/TTE showing the large vegetation on the atrial side of the mitral valve

**Figure 2:**
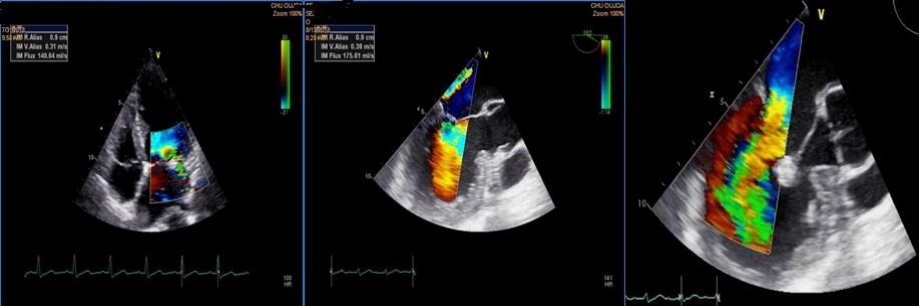
couple TTE/TTE showing a mitral regurgitation grade III

**Diagnostic assessment:** blood tests showed an elevation of inflammatory markers: white blood cell (WBC) at 19880/mm^3^, C reactive protein (CRP) at 153 mg/L and an elevated erythrocyte sedimentation rate (110 ml/min). Infective endocarditis was suspected; a couple transthoracic and transoesophageal echocardiography (TTE/TEE) showed a large vegetation on the atrial side of the mitral valve with a significant mitral regurgitation (Grade III) (Figure 1, Figure 2). Three Blood cultures were realized and have isolated a growth of *Streptococcus SPP*.

**Diagnosis:** a diagnosis of Infective endocarditis of *Streptococcus SPP* was made. The assessment of Infective endocarditis extension (Brain-chest-abdomen-pelvis CT, kidney function, dilated fundus exam) was without abnormalities.

**Therapeutic interventions:** an antibiotic therapy with amoxicillin (200 mg/kg/24h) was introduced for a month with good clinical and biological evolution (Disappearance of fever, WBC: 6800/mm^3^, CRP: 5 mg/l).

**Follow-up and outcome of interventions:** two months ago, the patient reported a pulsatile, non-painful swelling in the upper third of the right arm.

**Figure 3:**
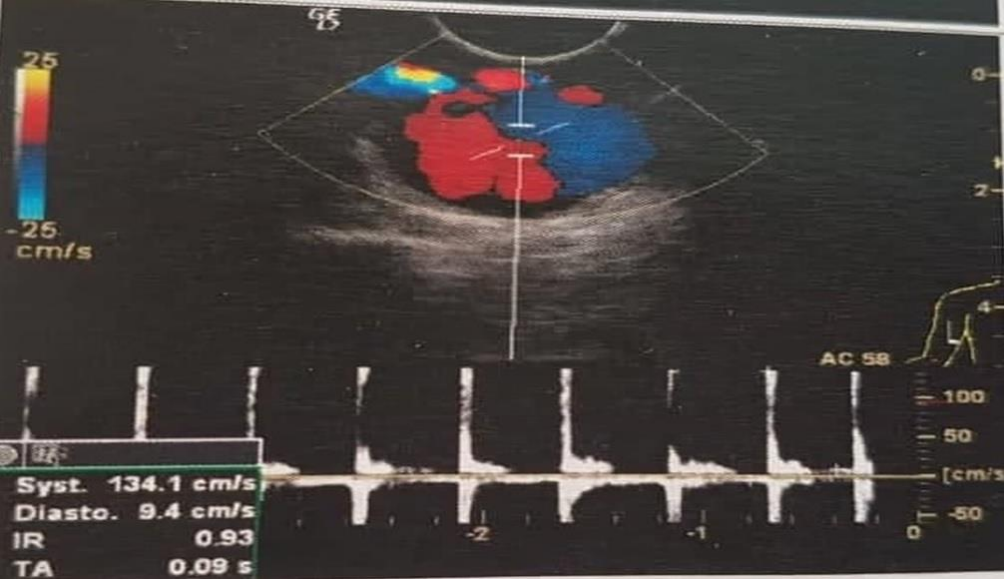
Doppler ultrasound of upper right limb showed the aspect of a sacciform aneurysm of brachial artery

**Diagnostic assessment:** Doppler ultrasound (DU) and CT scan showed a sacciform aneurysm of the right brachial artery (Figure 3).

**Figure 4:**
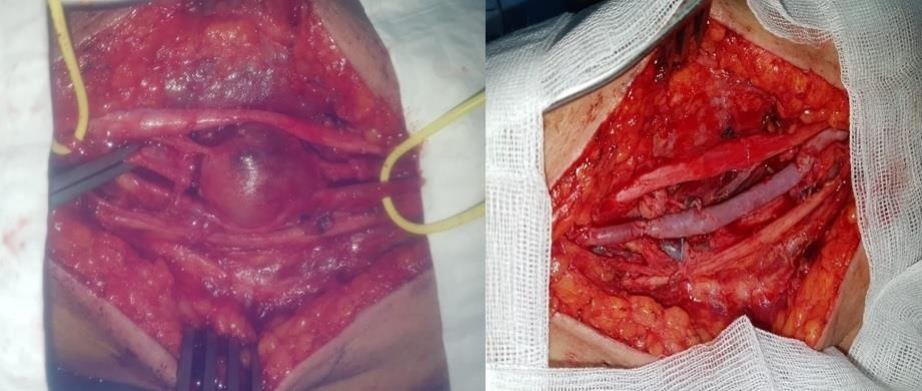
per-operatory images showing the aneurysm of the brachial artery and the venous graft after resection of the aneurysm

**Therapeutic interventions:** the patient underwent for a resection of the aneurysm then it was replaced by a segment of the great saphenous vein (Figure 4). Microbiological culture of the surgical specimen proved the same germ (*Streptococcus SPP*) and antibiotic was re-introduced with good evolution.

**Follow-up and outcome of interventions:** the patient had an excellent response to antibiotics. She remains asymptomatic and in excellent clinical condition.

**Informed consent:** the patient is consent for publication her case.

## Discussion

Infective endocarditis is a cardiovascular disease characterized by the inflammation of inner tissues of heart, the endocardium and usually of valves, which might be caused by a microbial agent especially bacteria, whereas the main pathogens are Streptococcus and Staphylococcus [1]. Infective endocarditis incidence is approximately 1.7-6.2 cases/100,000 patients per year [3]. Blood culture examination and Echocardiography (TTE/TEE) are the main means for making Infective endocarditis diagnosis, of course without forgetting the importance of cardiac CT and PET-scan when exist [3]. It can induce many cardiac and extra-cardiac complications; such as neurological, renal, vascular, musculoskeletal and sepsis. One complication in 57%, two in 26% and three or more in about 14% of patients, with a frequency depending on the infecting pathogen [4]. Congestive heart failure (CHF) and embolic complications are considered as the most important complications, with a great impact on prognosis. [4]

The inflammatory reaction against bacteria forms vegetation containing bacteria in valves, in the endocardium, and inner membrane blood vessels, then the dispersion of vegetation's fragments is responsible of various embolic complications [3]. Emboli may involve major arteries, mostly the central nervous system, but also other organs [2]. Usually, embolization develops during the first two weeks of therapy; it was reported that 76% of episodes occur before the initiation of antimicrobial therapy, and once adequate antibiotic therapy is initiated, the risk markedly decreases within two to three weeks [5]. Its incidence is higher in left-sided vegetation that are >1 cm, and varies from mitral to aortic vegetation (25% and 10% respectively) [6]. The effect of vegetation size on the risk of embolization is tightly depending on the germ. In streptococcus, the risk is volume-dependent. However, staphylococcal or fungal Infective endocarditis appears to have a high risk of embolization independently of vegetation´s size [7]. In our case, the responsible germ was *Streptococcus SPP* with vegetation´s size of (9*6 mm).

Mycotic aneurysms (MAs) are a rare complication of Infective endocarditis, which were described for the first time by Osler in 1885 [8]. They result from the diffusion of infection to the vessel wall and causes parietal ischemia that leads to degeneration of muscular wall, which induce an ectasia of the artery [9]. Mycotic aneurysm can touch all arterial territories; intracranial is the most commonly involved (48%-70%), aorta, visceral and rarely peripheral arteries [2]. Proximal location seems to be the most frequent [9], since that the peripheral ones are quite rare; 85% are because of intravenous drug use (IVDU), and only 9% are due to infective endocarditis. In our case, the patient presented with a MA of the brachial artery. The risk of mycotic aneurysm is directly correlated to the incidence of embolization, which means that they are more often observed in cases of mitral valve endocarditis, and especially when mitral vegetation are attached to the anterior leaflet [7], as was the case of our patient.

For proving the diagnosis, DU has the main place, sometimes supplemented by CT scan, which can precisely describe the lesion and its complications. Nevertheless, arteriography remains the reference for the choice of surgery [3]. The treatment of mycotic aneurysm complicating an Infective endocarditis consists two parts: the cure of valvulopathy ‘point of septic embolism’ and treatment of vascular ectasia. Antibiotic therapy adapted to the germ found in blood cultures is essential. It allows the sterilization of vegetations then avoiding bacteraemia and reinfection of a possible arterial prosthesis. Surgical intervention is the treatment of choice following a long course of antibiotics. In particular, it requires the eradication of infection by excision of the aneurysm and maintenance of adequate distal circulation. Endovascular repair using a stent-graft may also be used. Some studies have shown that an aggressive antibiotic therapy alone can reduce the size of mycotic aneurysm [10]. Early treatment of mycotic aneurysm can avoid the classic complications of rupture and acute thrombosis of the aneurysms but also the reinfection from an unsterilized aneurysmal spot during the treatment at the initial part of Infective endocarditis. However, the risk of reinfection and rupture of inter-posed vascular grafts is high [7].

The natural history of untreated mycotic aneurysm is fatal from either massive haemorrhage or fulminant sepsis [10]. In peripheral MAs, the rupture causes the death of approximately a quarter of patients and participates with acute thromboses in a high percentage of amputations [9]. In our reported case, the mycotic aneurysm was located at the brachial artery, and although it was possible to perform a brachial artery ligation, complete revascularization was preferred by using a saphenous vein graft.

## Conclusion

PMAs are extremely rare and severe complications of Infective endocarditis, but universally fatal without appropriate management. Diagnosis and surveillance remain clinical challenges, and CT angiography is currently considered the modality of choice. Treatment consists the excision of all intra-cardiac and/or extra-cardiac infected material and revascularization.
